# Applications of humic and fulvic acid under saline soil conditions to improve growth and yield in barley

**DOI:** 10.1186/s12870-024-04863-6

**Published:** 2024-03-15

**Authors:** Ibtisam Mohammed Alsudays, Fowzia Hamdan Alshammary, Nadiyah M. Alabdallah, Aishah Alatawi, Mashael M. Alotaibi, Khairiah Mubarak Alwutayd, Maha Mohammed Alharbi, Suliman M. S. Alghanem, Fahad Mohammed Alzuaibr, Hany S. Gharib, Mamdouh M. A. Awad-Allah

**Affiliations:** 1 Department of Biology, College of Science, Qassim University, Burydah, 52571 Saudi Arabia; 2 Department of Biology, College of Science, Imam Abdulrahman Bin Faisal University, Dammam, Saudi Arabia; 3 Department of Biology, College of Science, Imam Abdulrahman Bin Faisal University, P.O. Box 1982, Dammam, 31441 Saudi Arabia; 4 Basic & Applied Scientific Research Centre, Imam Abdulrahman Bin Faisal University, P.O. Box 1982, Dammam, 31441 Saudi Arabia; 5 Biology Department, Faculty of Science, University of Tabuk, Tabuk, 71421 Saudi Arabia; 6 Biology Department, College of Science and Humanities, Shaqra ‏University, Shaqra, Saudi Arabia; 7 Department of Biology, College of Science, Princess Nourah bint Abdulrahman University, P.O. Box 84428, Riyadh, 11671 Saudi Arabia; 8 Biology Department, Faculty of Science, University of Tabuk, Tabuk, 71491 Saudi Arabia; 9 Department of Biology, College of Science, Qassim University, Buraidah, Saudi Arabia; 10 Department of Biology, Faculty of Science, University of Tabuk, Tabuk, 47713 Saudi Arabia; 11 Department of Agronomy, Faculty of Agriculture, University of Kafrelsheikh, Kafrelsheikh, 33516 Egypt; 12 Field Crops Research Institute, Agricultural Research Center, Giza, 12619 Egypt

**Keywords:** Barley, Humic acid, Potassium humate, Fulvic acid, Salinity

## Abstract

**Background:**

Enriching the soil with organic matter such as humic and fulvic acid to increase its content available nutrients, improves the chemical properties of the soil and increases plant growth as well as grain yield. In this study, we conducted a field experiment using humic acid (HA), fulvic acid (FA) and recommended dose (RDP) of phosphorus fertilizer to treat Hordeum vulgare seedling, in which four concentrations from HA, FA and RDP (0.0 %, 50 %, 75 % and 100%) under saline soil conditions . Moreover, some agronomic traits (e.g. grain yield, straw yield, spikes weight, plant height, spike length and spike weight) in barley seedling after treated with different concentrations from HA, FA and RDP were determined. As such the beneficial effects of these combinations to improve plant growth, N, P, and K uptake, grain yield, and its components under salinity stress were assessed.

**Results:**

The findings showed that the treatments HA + 100% RDP (T1), HA + 75% RDP (T2), FA + 100% RDP (T5), HA + 50% RDP (T3), and FA + 75% RDP (T6), improved number of spikes/plant, 1000-grain weight, grain yield/ha, harvest index, the amount of uptake of nitrogen (N), phosphorous (P) and potassium (K) in straw and grain. The increase for grain yield over the control was 64.69, 56.77, 49.83, 49.17, and 44.22% in the first season, and 64.08, 56.63, 49.19, 48.87, and 43.69% in the second season,. Meanwhile, the increase for grain yield when compared to the recommended dose was 22.30, 16.42, 11.27, 10.78, and 7.11% in the first season, and 22.17, 16.63, 11.08, 10.84, and 6.99% in the second season. Therefore, under salinity conditions the best results were obtained when, in addition to phosphate fertilizer, the soil was treated with humic acid or foliar application the plants with fulvic acid under one of the following treatments: HA + 100% RDP (T1), HA + 75% RDP (T2), FA + 100% RDP (T5), HA + 50% RDP (T3), and FA + 75% RDP (T6).

**Conclusions:**

The result of the use of organic amendments was an increase in the tolerance of barley plant to salinity stress, which was evident from the improvement in the different traits that occurred after the treatment using treatments that included organic amendments (humic acid or fulvic acid).

**Supplementary Information:**

The online version contains supplementary material available at 10.1186/s12870-024-04863-6.

## Introduction

Barley (*Hordeum vulgare*) is one of the most important cereal crops in Egypt and the world. In addition, it is the fifth most productive cereal crop in the world. It is also one of the most adaptable cereal crops, as it has a diverse capability to adapt to different agricultural climatic conditions and diverse soil characteristics [1–3]. In terms of the nutritional importance, barley is the fourth most important food crop in Egypt as a grain crop. Its cultivated area reached 31,612 hectares in 2017, which produced a total yield of 115,478 tons with an average yield of 3.653 tons/ha [4]. Among the things that increase the great importance of barley is its capability to grow and produce in many marginal environments that are not suitable for the production of other grain crops such as wheat, which are often characterized by low temperatures, drought and salinity [5]. In 2017, the total area cultivated with barley in the world amounted to 47 million hectares, with a total production of 147.4 million tons per year and an average yield of 3.136 tons/ha [4]. There are many challenges facing agriculture in the world, including the need to increase food production by 70% to meet the continuous population increase, which is expected to increase with 2.3 billion by 2050. However, with the organization and economy in the consumption of natural resources and maximizing the use of them in a more efficient way and conserving them, and adapting to climate change as well as fighting poverty and hunger. Among the biggest of these challenges are also the problems of soil salinity and irrigation water, which are more severe in semi-arid regions, including Egypt [6].

Salinity results in several negative effects on the plant and its growth, as it reduces the rate of photosynthesis, decrease the uptake of vital nutrients for the plant and reduces the accumulation of dry matter [7–10]. One of the negative effects of salt accumulation in the soil is that it reduces the transport of water and ions from the soil to the plant [11]. Among the damages caused by salinity to the plant are also: a decrease in the growth rate and thus a decrease in the height of the plant, the number of leaves, buds, fresh and dry shoot weights and yield in the plants under salinity stress [10, 12, 13]. In addition, increasing the level of the salinity leads to a significant decrease in the leaf water contents, photosynthesis, growth, nutrient uptake, and plant productivity, the uptake of N, P and K by barley plants [14–17]. Increasing the concentration of sodium chloride (NaCl) in the irrigation water also led to a decrease in concentrations of magnesium, calcium and nitrogen in plant tissues [18]. Salinity stress causes a decrease in plant growth by negatively affecting biochemical reactions and many physiological processes such as photosynthesis, nutrient-mineral balance, antioxidant metabolism, osmolality accumulation, proline and hormonal signaling [19, 20].

One of the agriculturally, environmentally and economically sound practices is organic amendment, a method that has already been established and applied in several studies [21]. One of the advantages of using organic amendment is that it contributes in achieving agricultural sustainability, especially in intensive cropping systems. Organic amendment has gained paramount importance in recent years [8, 22]. Humic acid is one of the most active constituents of soil and is a major part of humus and organic matter [22]. Humic acid applications increase the organic carbon content, cation exchange capacity and pH of soil, [23]. The use of humic and fulvic acid results in improving the physical, biological and chemical properties of soil, which leads to an increased availability of nutrients for plants, improves fruit quality, improves soil fertility in an ecologically and environmentally way, stimulates and increases the activity of plant enzymes/hormones and reduces soil borne diseases, [24–31]. Organic amendments improve the chemical and physical properties of the soil, as they improve mineral nutrient status and improve plant growth plant productivity in saline soils by providing nutrients, especially N and P [8, 32–35]. One of the benefits of organic matter is that it improves soil properties and increase its water-holding capacity, and thus plays an important role in the soil ecosystem since it also provides substrates for microbial decomposition (which in turn provides mineral nutrients to plants) [32]. The application of HA treatment resulted in an increased plant growth, photosynthetic processes, antioxidant enzyme activity, dry weight, productivity and an increase in the content of N, P, K, Ca, Mg, Na, Fe, Zn, and Mn in plants grown under salinity stress compared to control [9, 36–38]. Whereas, is due to the fact that it greatly facilitates the transfer of elements from soil to the plant through increasing the permeability of the cell membrane [39]. Moreover, potassium (K) is known and classified as a macro fertilizer element and necessary for the majority physiological processes inside plants [40]. Where K plays an important and positive part in alleviating stresses such as drought and salinity in many crops [41–43].

Schnitzer and Khan, [44], showed that soil organic matter consists mainly of humic materials, as it often constitutes about 60 to 70% of the total organic matter. A benefit of humic acid treatment is that it can decrease the segregation effects of periodic wetting and drying on soil structural stability [45, 46]. In addition, it works to a large extent to maintain soil porosity, which is an vital component of soil structure and productive capacity [26, 46]. Previous studies showed some of the factors that have a significant impact on the appearance of the effects of humic substances on plant growth and plant productivity, including the weight of the molecular fraction. Where the low humic molecular size fraction is preferred because it easily reaches the plasma lymph in plant cells, which leads to a positive effect on plant growth, and this is due to the uptake of nutrients, especially nitrates. However, until now its effects on intermediate metabolites are still not understood, but it appears that humic substances may affect both respiration and photosynthesis [26]. Nardi et al., [26] demonstrated that the stimulatory effects of humic substances were associated directly to enhanced uptake of macronutrients such as nitrogen (N), phosphorus (P) and sulfur (S) as well as micronutrients such as iron (Fe), zinc (Zn), copper (Cu) and manganese (Mn). The effects of applying humic and fulvic acids to crops are usually associated with enhanced root initiation and improved root growth [47]. It became clear from the many studies thar investigated humic acid and fulvic acids and their effect on plants that it is an organically charged bio-stimulant that greatly and significantly affects plant growth and increases the productivity and grain yield [48].

The current study aimed to investigate the effect of integration between humic acid or fulvic acid with different rates of phosphorus on the yield and its components of barley under saline soil conditions, in order to reduce the harmful effects of salinity and study the extent to which it is possible to reduce the application rates of phosphorus fertilizer and increase plant yield.

The research hypothesis was that the addition of humic or fulvic acid in addition to different rates of phosphate fertilization with mono-superphosphate would have the ability to reduce the harmful effect of salinity and improve growth and yield in barley under saline soil conditions.

## Materials and methods

### Site description

Two field experiments were conducted during the winter seasons of 2018/2019 and 2019/2020 at the Sakha Production Sector Farm, Agricultural Research Center, Kafr El-Sheikh Governorate, Egypt (31° 06' N latitude, 30° 56' E longitude) to study the effect of treatments consisting of combinations of ratios of the recommended rate of phosphorus fertilizers and humic acid (potassium humate) or fulvic acid on some agronomic characteristics, yield and its components, as well as the uptake of nutrients from barley cultivar (*Hordeum vulgare*, L. c.v. Giza 123) under saline soil conditions. The soil classification of the study site was saline-sodic clay, and Table 1 shows the different characteristics of the soil of the study site (0-30 cm of surface), according to the USA soil classification [49]. As for the average monthly climatic data for the site during the 2018/2019 and 2019/2020 barley growing seasons, it is shown in Table S1. Table 2 shows some chemical properties and compositions of humic (HA) and fulvic (FA) acid used in the experiment.

**Table 1:** The physical and chemical properties for the soil of study site

**Season**	**Physical Property**			**Chemical Property**										
	**Sand%**	**Silt%**	**Clay%**	**pH**	**EC (dS m**^**−1**^**)**	**SAR**	**ESP**	**Soluble Cation** **(meq 100 g**^**−1**^** soil)**				**Soluble Anions (meq 100 g**^**−1**^** soil)**		
								**Na**^**++**^	**K**^**+**^	**Ca**^**++**^	**Mg**^**++**^	**HCO3**^**-**^	**Cl**^**-**^	**SO4**^**--**^
2018/2019	28.34	23.45	48.21	8.21	10.53	18.64	42.23	43.40	1.14	9.86	29.63	58.30	40.90	14.30
2019/2020	25.32	26.44	48.24	8.22	10.65	18.76	42.21	43.70	1.15	9.88	29.65	58.60	40.30	14.60

**Table 2:** Some chemical properties and compositions of humic (H) and fulvic (F) acid used in the experiment

**Characteristics**	**Humic acid**	**Fulvic acid**
pH	5.64	1.58
Ec (dsm-1)	0.10	0.11
**Humic acid**	85%	K fulvate 85%
**Fulvic acid**	3%	
K_2_O	12%	10%
K	10%	10%
Organic N	1%	0.38
Available N (ppm)	1.72	0.54
Available P (ppm)	0.23	0.17
Available K (ppm)	1.95	1.87

### Experimental design and management

The experiment was designed and set up in a Randomized Complete Block Design (RCBD) with 12 treatments in three replications. The treatments included twelve combinations of humic acid (HA) at rate of 4.75 kg/ha or fulvic acid (FA) at rate of 4.75 L/ha or without adding (control), with four ratios of recommended dose (RDP) of phosphorus fertilizer. The RDP fertilizer was 53.57 kg P_2_O_5_ ha^-1^ as 357.14 Kg of calcium superphosphate (15.5% P_2_O_5_). The area of the experimental plot was 15 m^2^ (5 x 3 m), consisting of 15 rows, each row separated by 0.20 m. Nitrogen (N) fertilizer was added 142.86 kg N/ha in the form of ammonium nitrate (33.5% N) in three equal portions where the first part was added after 21 days of sowing (DAS), the second part was added at 35 days, while the third and last part was added at 50 days. Concerning the potassium fertilizer, the first dose was added with the first dose of nitrogen fertilizer. Barley was sown on December 1^st^ for both seasons, and the seed rate used was 119 kg/ha. Sowing was done after rice as a preceding crop in both seasons. As for all other agricultural practices, they were conducted in accordance with the technical recommendations for barley cultivation according to Egyptian Ministry of Agriculture recommended fields.

### Studied traits

Before harvesting and 120 days after sowing, ten plants per plot were randomly selected to measure the plant height (cm), the length of the spike (cm), the weight of the spike (g), the weight of 1000 grains (g) and the number of grains per spike. All plants on each experimental plot were then harvested and separated into straw and grain to determine and estimate straw and grain yield per hectare. Grain and straw samples were then obtained from all experimental units, dried at 65 °C to constant weight, and then pounded. Proline content was determined according to Bates et al. [50]. The micro-Kjeldahl method according to AOAC [51] was used to estimate the total N in straw and grain. While the colorimetrically using chlorostannous decreased molybdophosphoric blue color method as described by Chapman and Parker [52] was used to estimate the phosphorous content (P%). As for the determination of the potassium content (K%) in the digested plant materials, it was done using a flame photometer according to Page et al., [53]. As for the determination of the uptake of N, P and K (kg ha^-1^), it was done by multiplying the yield of grain or straw with its content N%, P% and K%, respectively.

### Statistical analysis

The analysis of variance (ANOVA) of randomized complete block design as mentioned by Casella [54] was used to analyze the data statistically, using Costat software program Version 6.303 [55]. Duncan’s multiple range test at 0.05 level of probability by Waller and Duncan [56] was used for comparing treatment means.

## Results

### Agronomic, yield and its components

The application of the four treatment combinations, which consist of different ratios of RDP fertilizer and humic acid or fulvic acid, resulted in significant effects on the agronomic traits, yield and its components in barley compared to the control (without adding) in each of the 2018/2019 and 2019/2020 seasons.

**Fig. 1:**
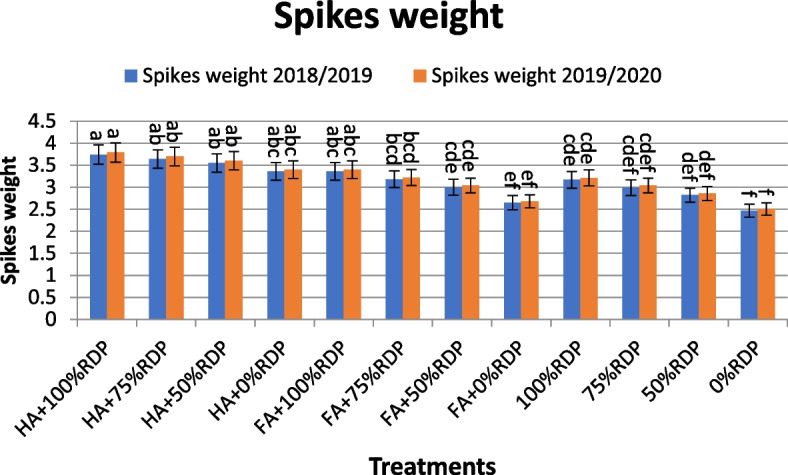
The effects of treatment with humic acid or fulvic acid and different rates of the recommended dose of phosphorus fertilizer (RDP) on spikes weight (g) of barley cultivar Giza 123 in the seasons 2018/2019 and 2019/2020. Different letters associated values indicate significant differences between values at *p*≤ 0.05 according to Duncan's multiple range test

**Fig. 2:**
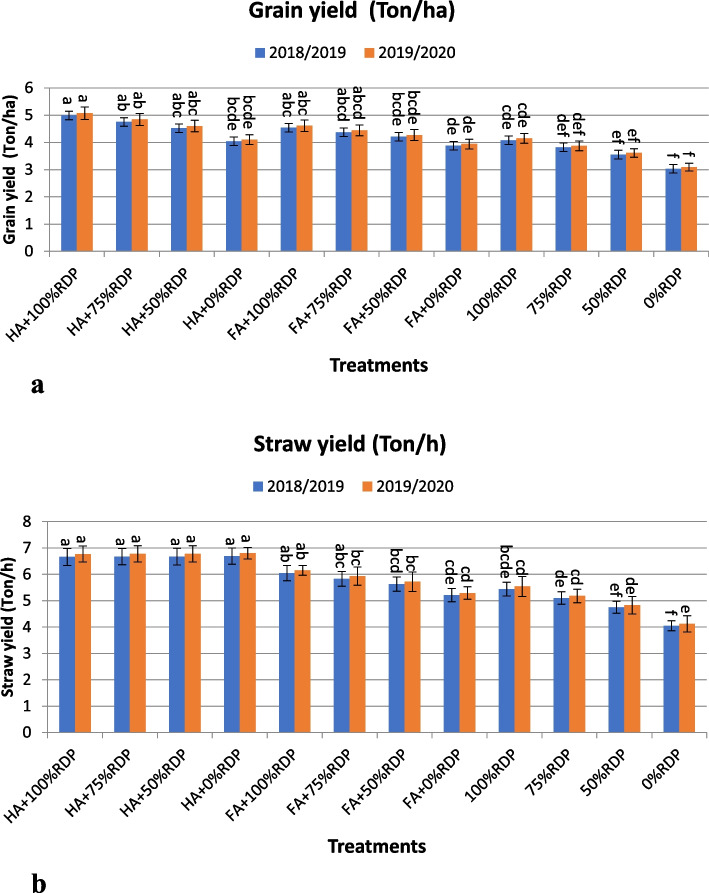
**a** and **b**. The effects of treatment with humic acid or fulvic acid and different rates of the recommended dose of phosphorus fertilizer (RDP) on grain and straw yield (Ton/ha) of barley cultivar Giza 123 in the seasons 2018/2019 and 2019/2020. Different letters associated values indicate significant differences between values at *p*≤ 0.05 according to Duncan's multiple range test

The highest values for most of the traits under study were recorded as a result of the application of treatments containing HA+100%RDP, HA+75%RDP and FA+100%RDP, FA+75%RDP (Tables 3, 4, 5, and 6, Figs. 1 and 2a and b). The results in Table 3 showed that there were significant effects on plant height and spike length obtained as a result of applying the treatments in both studied seasons. In this direction, the treatments; T5 (FA + 100% RDP), T6 (FA + 75% RDP), T7 (FA + 50% RDP), and T1 (HA + 100% RDP) , recorded the highest values of plant height with increased percentage compared the same phosphorus application (T9, T10 and T11) 6.09%, 7.57%, 7.10%, and 0.96% , at the second season.

**Table 3:** The effects of treatment with humic acid or fulvic acid and different rates of the recommended dose of phosphorus fertilizer (RDP) on plant height, and spike length of barley cultivar Giza 123 in the seasons 2018/2019 and 2019/2020

**Treatments**	**Plant height (cm)**		**Spike length**	
	**2018/2019**	**2019/2020**	**2018/2019**	**2019/2020**
HA + 100% RDP	109.72 ± 0.66 ab	113.59 ± 2.12 bc	10.76 ± 0.52 a	10.96 ± 0.21 a
HA + 75% RDP	107.51 ± 2.15 bc	111.30 ± 2.08 bc	10.55 ± 0.21 a	10.75 ± 0.21 ab
HA + 50% RDP	105.30 ± 0.60 bcd	109.01 ± 2.03 cd	10.35 ± 0.21 ab	10.54 ± 0.49 abc
HA + 0% RDP	100.92 ± 0.50 d	104.44 ± 1.95 d	9.93 ± 0.27 abc	10.12 ± 0.32 abcd
FA + 100% RDP	115.30 ± 2.02 a	119.36 ± 1.10 a	9.74 ± 0.35 abcd	9.93 ± 0.46 abcd
FA + 75% RDP	112.75 ± 1.91 ab	116.72 ± 1.93 ab	9.65 ± 0.46 abcd	9.84 ± 0.37 abcd
FA + 50% RDP	110.20 ± 0.88 ab	114.07 ± 2.00 ab	9.56 ± 0.45 abcd	9.75 ± 0.45 abcd
FA + 0% RDP	105.09 ± 2.09 cd	108.79 ± 1.97 c	9.38 ± 0.45 abcd	9.56 ± 0.44 abcd
100% RDP	108.68 ± 2.65 bcd	112.51 ± 3.84 ab	9.15 ± 0.43 bcde	9.32 ± 0.43 bcd
75% RDP	106.75 ± 2.33 abc	108.51 ± 3.33 c	8.93 ± 0.43 bcde	9.10 ± 0.42 cd
50% RDP	104.85 ± 3.28 cd	106.51 ± 1.91 c	8.71 ± 0.41 cde	8.88 ± 0.41 cd
0% RDP	100.96 ± 1.55 d	102.51 ± 1.45 d	8.28 ± 0.39 de	8.43 ± 0.39 d

The values are the mean values ± the standard error. Different letters associated values indicate significant differences between values at *p*≤ 0.05 according to Duncan's multiple range test

**Table 4:** The effects of treatment with humic acid or fulvic acid and different rates of the recommended dose of phosphorus fertilizer (RDP) on number of grain per spikes and 1000-grain weight of barley cultivar Giza 123 in the seasons 2018/2019 and 2019/2020

**Treatments**	**No. of grain/spike**		**1000-grain weight**	
	**2018/2019**	**2019/2020**	**2018/2019**	**2019/2020**
HA + 100% RDP	57.10 ± 2.71 a	57.88 ± 2.63 a	58.40 ± 1.30 a	59.17 ± 1.04 a
HA + 75% RDP	55.23 ± 2.62 ab	56.00 ± 2.55 ab	56.93 ± 1.53 ab	57.68 ± 0.97 ab
HA + 50% RDP	53.38 ± 2.53 ab	54.11 ± 2.46 abc	55.46 ± 1.18 abc	56.19 ± 3.07 a
HA + 0% RDP	49.65 ± 2.36 abcd	50.34 ± 2.29 abcde	52.52 ± 2.41 ab	53.21 ± 1.08 cde
FA + 100% RDP	54.34 ± 2.58 ab	55.09 ± 2.50 abc	54.17 ± 2.57 bc	54.88 ± 1.58 bc
FA + 75% RDP	51.65 ± 2.45 abc	52.36 ± 2.38 abcd	53.39 ± 1.06 bc	53.66 ± 1.07 cd
FA + 50% RDP	48.96 ± 2.32 abcd	49.64 ± 2.26 bcdef	51.75 ± 1.73 cd	52.43 ± 1.09 cdef
FA + 0% RDP	43.58 ± 2.07 de	44.18 ± 2.01 fg	49.32 ± 1.52 d	49.97 ± 0.92 fg
100% RDP	49.38 ± 2.35 bcd	50.06 ± 2.27 cdef	53.05 ± 2.20 abc	53.75 ± 1.07 cd
75% RDP	46.82 ± 2.22 cde	47.47 ± 2.16 defg	51.55 ± 1.72 bc	52.22 ± 2.38 def
50% RDP	44.27 ± 2.10 de	44.88 ± 2.04 efg	50.04 ± 0.70 cd	50.70 ± 0.94 efg
0% RDP	39.17 ± 1.86 e	39.71 ± 1.80 g	47.04 ± 1.58 d	47.65 ± 1.18 g

The values are the mean values ± the standard error. Different letters associated values indicate significant differences between values at *p*≤ 0.05 according to Duncan's multiple range test

**Table 5:** The effects of treatment with humic acid or fulvic acid and different rates of the recommended dose (RDP) of phosphorus fertilizer on biological yield (Ton/ha) and harvest index of barley cultivar Giza 123 in the seasons 2018/2019 and 2019/2020

**Treatments**	**Biological yield**		**harvest index**	
	**2018/2019**	**2019/2020**	**2018/2019**	**2019/2020**
HA + 100% RDP	11.65 ± 0.69 a	11.85 ± 0.54 a	42.77 ± 0.66 a	42.83 ± 0.02 abcd
HA + 75% RDP	11.42 ± 0.67 a	11.61 ± 0.53 ab	41.54 ± 0.73 ab	41.67 ± 0.00 d
HA + 50% RDP	11.19 ± 0.53 ab	11.37 ± 0.51 ab	40.36 ± 0.01 c	40.41 ± 0.03 e
HA + 0% RDP	10.73 ± 0.50 abc	10.90 ± 0.49 ab	37.65 ± 0.01 d	37.60 ± 0.48 f
FA + 100% RDP	10.59 ± 0.50 abc	10.76 ± 0.49 abc	42.88 ± 0.03 ab	42.82 ± 1.13 cd
FA + 75% RDP	10.21 ± 0.49 abcd	10.38 ± 0.47 abc	42.84 ± 0.03 ab	42.88 ± 1.07 a
FA + 50% RDP	9.83 ± 0.47 bcde	10.00 ± 0.46 bcd	42.77 ± 0.81 b	42.79 ± 0.59 abc
FA + 0% RDP	9.09 ± 0.43 def	9.24 ± 0.42 de	42.71 ± 0.04 ab	42.70 ± 0.04 bcd
100% RDP	9.52 ± 0.46 cdef	9.68 ± 0.44 cde	42.83 ± 0.01 ab	42.91 ± 0.64 abc
75% RDP	8.91 ± 0.43 ef	9.06 ± 0.41 de	42.83 ± 0.03 ab	42.78 ± 0.63 abc
50% RDP	8.30 ± 0.39 fg	8.44 ± 0.38 ef	42.73 ± 0.02 ab	42.88 ± 0.67 ab
0% RDP	7.08 ± 0.34 g	7.20 ± 0.33 f	42.82 ± 0.04 ab	42.94 ± 0.88 ab

The values are the mean values ± the standard error. Different letters associated values indicate significant differences between values at *p*≤ 0.05 according to Duncan's multiple range test

**Table 6:** The effects of treatment with humic acid or fulvic acid and different rates of the recommended dose of phosphorus fertilizer (RDP) on the uptake of N, P, and K in straw (kg/ha) of barley cultivar Giza 123 in the seasons 2018/2019 and 2019/2020

**Treatments**	**Uptake N (kg/ha)**		**Uptake P (kg/ha)**		**Uptake K (kg/ha)**	
	**2018/2019**	**2019/2020**	**2018/2019**	**2019/2020**	**2018/2019**	**2019/2020**
HA + 100% RDP	101.99 ± 4.84 a	103.33 ± 2.96 a	16.20 ± 0.77 a	16.42 ± 0.76 a	41.94 ± 1.99 a	42.49 ± 1.96 a
HA + 75% RDP	93.17 ± 4.42 ab	94.41 ± 1.56 b	15.17 ± 0.72 ab	15.37 ± 0.71 ab	36.97 ± 1.75 b	37.45 ± 1.73 b
HA + 50% RDP	84.36 ± 4.00 bc	85.47 ± 1.06 bc	14.12 ± 0.67 bc	14.31 ± 0.65 bc	31.99 ± 1.52 cd	32.41 ± 1.50 cd
HA + 0% RDP	66.73 ± 3.17 ef	67.61 ± 1.12 e	12.05 ± 0.57 def	12.21 ± 0.57 def	22.03 ± 1.05 g	22.32 ± 1.03 g
FA + 100% RDP	85.51 ± 4.06 bc	86.62 ± 2.27 c	13.71 ± 0.65 bcd	13.88 ± 0.64 bcd	34.30 ± 1.63 bc	34.75 ± 1.60 bc
FA + 75% RDP	78.13 ± 3.71 cd	79.16 ± 2.46 d	12.97 ± 0.62 cde	13.14 ± 0.60 cde	31.00 ± 1.47 cde	31.40 ± 1.45 cd
FA + 50% RDP	70.77 ± 3.36 de	71.69 ± 2.59 d	12.23 ± 0.58 cde	12.39 ± 0.57 de	27.70 ± 1.31 de	28.07 ± 1.29 de
FA + 0% RDP	56.02 ± 2.66 gh	56.75 ± 1.80 f	10.77 ± 0.51 fg	10.91 ± 0.50 fg	21.12 ± 1.01 g	21.39 ± 0.98 g
100% RDP	64.75 ± 3.07 efg	65.61 ± 3.03 ef	11.79 ± 0.56 efg	11.95 ± 0.55 efg	28.01 ± 1.33 ef	28.39 ± 1.31 ef
75% RDP	58.63 ± 2.78 fgh	59.40 ± 2.74 fg	10.12 ± 0.48 gh	10.26 ± 0.47 gh	24.21 ± 1.15 fg	24.54 ± 1.13 fg
50% RDP	52.50 ± 2.49 h	53.19 ± 2.46 g	8.46 ± 0.40 h	8.57 ± 0.40 h	20.42 ± 0.97 g	20.69 ± 0.96 g
0% RDP	40.26 ± 1.91 i	40.79 ± 1.88 h	5.12 ± 0.24 i	5.19 ± 0.24 i	12.81 ± 0.61 h	12.99 ± 0.60 h

The values are the mean values ± the standard error. Different letters associated values indicate significant differences between values at *p*≤ 0.05 according to Duncan's multiple range test

Concerning spike length, the treatments T1 (HA + 100% RDP), T2 (HA + 75% RDP), T3 (HA + 50% RDP), and T4 (HA + 0% RDP), gave the highest mean values (10.76, 10.55, 10.35 and 9.93) in the first year, Table 3, with increased percentage compared the same phosphorus application (T9, T10, T11, and T12) 17.60%,18.14%, 18.83%, and 19.93%, respectively. In the same time, the treatments T1 (HA + 100% RDP), T2 (HA + 75% RDP), T3 (HA + 50% RDP), and T4 (HA + 0% RDP), recorded the highest mean values (10.96, 10.75, 10.54, and 10.12) in the second season, Table 3, with increased percentage compared the same phosphorus application (T9, T10, T11, and T12) 17.60%,18.13%, 18.69%, and 20.05%, respectively.

The highest spike weight values were obtained by applying the combinations T1 (HA + 100% RDP), T2 (HA + 75% RDP), T3 (HA + 50% RDP), and T4 (HA + 0% RDP), Fig. 1, with the increase over the same phosphorus application with a percentage of 17.98%, 21.74%, 25.89%, and 36.03%, in the first year and 18.07%, 21.71%, 25.87%, and 35.46%, in the second year, respectively.

The greatest increase percentage in number of grain/spike was recorded by applying the treatment T1 (HA+100% RDP) by 15.63% and 15.62% as well as the combination T2 (HA+75% RDP) by 17.96% and 17.97% compared with the same phosphorus application T9 and T10, , respectively, Table 4. The treatment T1 (HA+100% RDP) showed the heaviest weight of 1000-grain and significantly followed by the combinations T2 (HA+75% RDP), T3 (HA+50% RDP), and T5 (FA+100% RDP), with an increase over the same phosphorus application in the weight of 1000-grain by 10.08%, 10.44%, 10.83% and 2.11%, in the first season, while the percentage increase over the control in the second season was 10.08%, 10.46%, 10.83% and 2.10%, (Table 4).

For grain yield/ha (Fig. 2a), the combination treatments of T1 (HA + 100% RDP), T2 (HA + 75% RDP), T5 (FA + 100% RDP), T3 (HA + 50% RDP), T6 (FA +75% RDP) recorded the highest grain yield (Fig. 2a). The application of treatment T1 recorded an increase by 22.30 and 22.17% of grain yield compared to the same phosphorus application in the two years, respectively. Moreover, the application of T2 treatment showed a significant increase by 24.35 and 25.06% in grain yield compared with the same phosphorus application in the two study seasons, respectively. While the increase over the RDP was 16.42 and 16.63% in both seasons, respectively. Also, T5 recorded significantly increased by 11.27 and 11.08 % in grain yield over the same phosphorus application (the recommended dose) at the two years, respectively. Besides, T3 (HA + 50% RDP) significantly superior grain yield over control by 27.32 and 27.42 %, at the two studied seasons, respectively. But, the increase was 10.78, and 10.84 %, over the recommended dose at the both seasons, respectively (Fig. 2a). Finally, the application of combination of T6 (FA + 75% RDP), resulted to increase grain yield by 14.40 and 14.73 % over the same phosphorus application, while the increase was 7.11, and 6.99 % compared with the recommended dose at the two consecutive seasons, respectively.

On the contrary, the application of humic acid treatments; T1 (HA + 100% RDP), T2 (HA + 75% RDP), T3 (HA + 50% RDP), T4 (HA + 0% RDP), recorded the maximum yield of straw without significant differences between these treatments in the two studied seasons, with increase percentage compared with the same phosphorus application 22.43, 30.78, 40.42, and 65.19 % in the first season and 22.20, 30.89, 40.37, and 65.05 % in the second season, respectively (Fig. 2b). While, the application of folvic treatments T5 (FA + 100% RDP), and T6 (FA + 75% RDP), resulted to increase in straw yield compared with the same phosphorus application by percentage 11.21 and 11.01 % in the 2018/2019 season and 14.31 and 14.48 % in the 2019/2020 season, respectively.

The data shown in Table 5 indicated that biological yield affected by the combination among HA or FA, and percentage of RDP fertilizer on barley. The treatment T1 (HA+100% RDP) recorded the highest values of biological yield in the two studied seasons, with increased percentage of; 22.37 and 22.42% in the both seasons, respectively, over the same phosphorus application.. Also, the combinations treatments; T2 (HA + 75% RDP), T3 (HA + 50% RDP), T4 (HA + 0% RDP), T5 (FA + 100% RDP), and T6 (FA + 75% RDP), recorded highest values after T1, in both seasons, respectively.

From the results obtainable in Table 5 showed no significant differences for harvest index and no affected by the combination among humic acid, fulvic acid and percentage of RDP fertilizer on barley.

#### Physiological parameter analysis

**Fig. 3:**
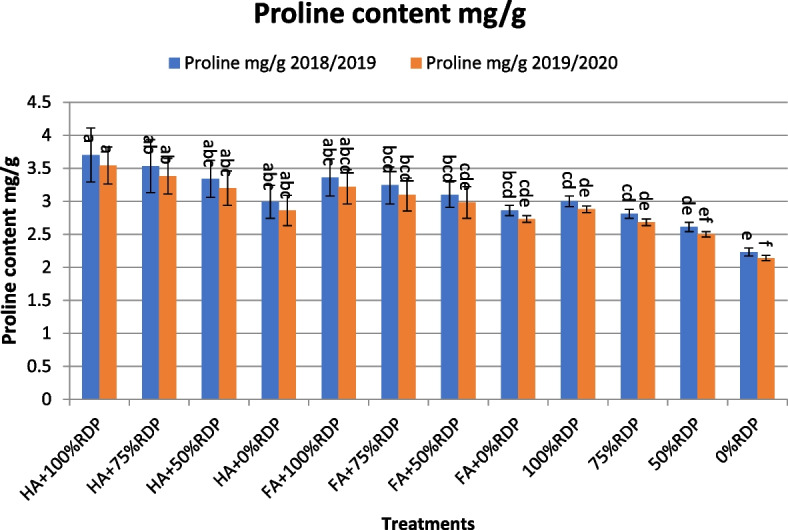
The effects of treatments with humic acid or fulvic acid and different rates of the recommended dose of phosphorus fertilizer (RDP) on proline content (mg/g) of barley cultivar Giza 123 in the seasons 2018/2019 and 2019/2020. Different letters associated values indicate significant differences between values at *p*≤ 0.05 according to Duncan's multiple range test

High values of proline content were found by applied T1 followed by applied T2 next T5 and T3, respectively, at the first and second year, (Fig. 3). From the results, it is clear that proline accumulation increases in plants under all treatments, which leads to increased plant tolerance to salinity stress.

### Nutrients uptake in grain and straw in barley

**Fig. 4:**
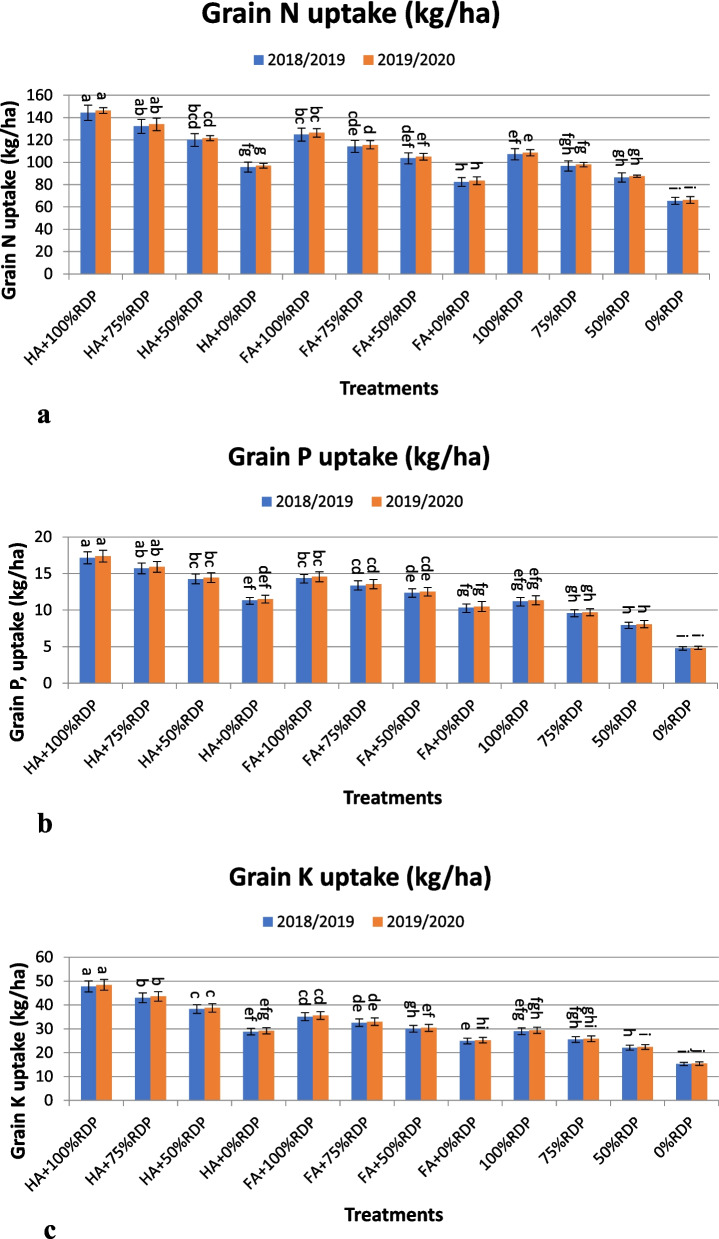
(**a**, **b** and **c**). The effects of treatment with humic acid or fulvic acid and different rates of the recommended dose of phosphorus fertilizer (RDP) on the uptake of N, P, and K in grain (kg/ha) of barley cultivar Giza 123 in the seasons 2018/2019 and 2019/2020. Different letters associated values indicate significant differences between values at *p*≤ 0.05 according to Duncan's multiple range test

The application of combinations between humic or fulvic and percentage of phosphorus fertilizer in the seasons 2018/19 and 2019/20 resulted in obtaining statistically significant differences in the content of grains of N, P, and K, (plant uptake of these elements) (Fig. 4a, b and c). The treatments T1 (HA + 100% RDP), T2 (HA + 75% RDP), T5 (FA + 100% RDP), T3 (HA + 50% RDP), and T6 (FA + 75% RDP), gave the highest value of N uptake in grain, however the treatment T12 (0% RDP) obtained the lowest values, with the percentage increase over the same phosphorus application (T9, T10, T9, T11 and T10) were; 34.70, 36.64, 16.41, 39.07, and 18.03% in the first year (Fig. 4a), however, the increase percentage were; 34.69, 36.66, 39.06, 16.42, and 18.04 % in the second year, respectively.

In addition, the treatments T1 (HA + 100% RDP), T2 (HA + 75% RDP), T5 (FA + 100% RDP), T3 (HA + 50% RDP), T6 (FA + 75% RDP), and T7 (FA + 50% RDP), gave the highest value of P uptake in grain in both seasons, Fig. 4b. The increase compared to same phosphorus application T9, T10, T9, T11, and T10; was in percent; 53.17, 63.78, 28.42, 78.80, 39.46, and 54.96 %, in the first year, while, the increase percentage were; 53.17, 63.92, 28.40, 78.81, 39.59, and 55.14 % in the second year, respectively.

Furthermore, the uptake of K in grain recorded the highest values by applying the treatments; T1 (HA + 100% RDP), T2 (HA + 75% RDP), T3 (HA + 50% RDP), T5 (FA + 100% RDP), and T6 (FA + 75% RDP), in two studied seasons, Fig. 4c. With increase over the same phosphorus application T9, T10, T11, T9, and T10 by percentage was; 64.85, 68.47, 73.12, 21.13, and 27.42 %, in the first year, while, the increase percentage were; 64.80, 68.46, 73.11, 21.09, and 27.44 %, in the second year, respectively.

The application of the studied treatments fertilizer rate combinations of humic acid, fulvic acid and phosphorus resulted in significant differences among them in the content of N, P and K, in straw in the seasons 2018/19 and 2019/20 (Table 6).

The combinations; T1 (HA + 100% RDP), T2 (HA + 75% RDP), T5 (FA + 100% RDP), T3 (HA + 50% RDP), T6 (FA + 75% RDP), T4 (HA + 0% RDP), and T7 (FA + 50% RDP), recorded the highest values of nitrogen uptake in straw and had a percentage increases compared to same phosphorus application (T9, T10, T9, T11, T10, T12 and T11) were; 57.51, 58.91, 32.06, 60.69, 33.26, 65.75, and 34.80%, at the first seasons and 57.49, 58.94, 32.02, 60.69, 33.27, 65.75, and 34.78 %, at the second year, respectively, Table 6.

The treatment T1 (HA + 100% RDP), T2 (HA + 75% RDP), T3 (HA + 50% RDP), T5 (FA + 100% RDP), and T6 (FA + 75% RDP), recorded the highest value of the uptake of P, in straw, however the treatment T12 (0% RDP) showed the lowest value, Table 6. Concerning the percentage increase over the same phosphorus application treatments (T9, T10, T11, T9, and T10) were; 37.40, 49.90, 66.90, 16.28, and 28.16%, in the first year, while, the increase percentage was 37.41, 49.81, 66.98, 16.15, and 28.07% in the second year, respectively (Table 6).

As a final point, the treatments; T1 (HA + 100% RDP), T2 (HA + 75% RDP), T5 (FA + 100% RDP), T3 (HA + 50% RDP), and, T6 (FA + 75% RDP), achieved the highest values of straw K uptake in both seasons. In the meantime, the treatment T12 (0% RDP) showed the lowest value of uptake of K in straw, Table 6. With the increase higher than the same phosphorus application treatments (T9, T10, T9, T11, and T10) increased by; 49.73, 52.71, 22.46, 56.66, and 28.05% in the first year, however, the increase percentage was; 49.67, 52.61, 22.40, 56.65, and 27.95 % in the second year, respectively.

## Discussion

### Agronomic and yield attributed traits

There are many negative effects of salinity stress on the plant. It in general decreases plant growth through affecting physiological processes for example photosynthesis by reducing the content in photosynthetic pigments in leaves, hormones, and enzyme activities, which leads to a significant decrease in grain yield in barley under salinity conditions, [2, 57–60]. This decrease in growth is most likely due to some factors, for instance osmotic pressure, ionic toxicity, limitation of nutrient uptake, decreased photosynthetic processes, and accumulation of sodium in plant tissues [47, 61, 62]. In this study, treatment with potassium humate (HA) or FA increased plant growth rate and its parameters under salinity compared with the control group, (Fig. 2a and b).

The application of combinations T1, T2, T3, T5, followed by T6 and T7 showed the highest values for the studied traits (Tables 4  and 5, Figs. 1 and 2). The treatments by HA and FA significantly improved agronomic traits, yield and its components compared the control (without HA or FA) treatment. In this regard, it is possible to increase the nutrients available in the soil, improve the chemical properties of soil and thus increase plant growth and productivity by enriching soil with organic modifiers such as adding humic acid or fulvic acid, [41, 63]. In this study, the treatments consisting of combinations between four different percentages of the RDP fertilizer and humic or fulvic acid affected the agronomic traits, yield components and barley yield significantly compared with the control treatment (without addition) in the 2018/2019 and 2019/2020 seasons (Figs. 1, 2, 3 and 4).

Under the conditions of saline soils, the treatment with organic amendments (whether humic acid or fulvic acid) recorded in a increase in the traits values of barley plants over the control treatment or compared to the recommended dose of phosphorous fertilizer (RDP), while the treatments; T5 (FA + 100% RDP), T6 (FA + 75% RDP), and T7 (FA + 50% RDP), increased plant height by 16.44%, 13.86% and 11.28%, respectively, above the control (T12). The treatment by adding HA recorded in a major increase in plant height, [64]. The humic substances (humic and fulvic acids), humic substances significantly increased the yield and its components relative to without humic substances treatment. Also, humic acid was superior to fulvic acid or without humic substances. These enhancements may be due to humic substances, which are the main constituents (65-70%) of soil organic matter, and which greatly increase plant growth for several reasons including increased cell membrane permeability, oxygen and phosphorus uptake, respiration, photosynthesis, and growth supplying root cells. A distinct effect of humic acid was observed among plants. Many previous studies on various crops reported the positive and stimulating effect of HA on growth, yield increase and increase of nutrient uptake [65–68]. The use of humic acid or FA results in several effects, including direct or indirect effects. The indirect effects happen mostly through properties such as: soil nutrient enrichment, increased cation exchange capacity (CEC), increased microbial population, and improved soil structure; While the direct effects are blunt about a variety of biochemical actions exerted on membrane, the cell wall or cytoplasm and are mainly of a hormonal nature, the hormone-like activities of humic acid have been well documented in many studies, particularly auxins, gibberellins and cytokinins [68–73]. The reason is due to the positive effect of organic modifications on yields through different mechanisms, which are not mutually exclusive, including the supply of mineral nutrients [74], and the use of treatment with some organic modifications resulted in a significant improvement in growth parameters and yield of barley plants significantly, [41, 75].

The organic matter plays a large and important function in the soil ecosystem as it provides substrates for microbial decomposition (which in turn supplies mineral nutrients to plants as they become available to plants), and improves water-holding capacity and soil structure [38, 41, 76]. This is explained by the fast availability of nutrients, particularly the patterns of nitrogen (N) release and mineralization kinetics. The positive function of Kh (HA) in promoting plant growth is due to its role in increasing the organic matter of the growth media, which leads to increased water retention and availability, conserved mineral nutrient and availability preserved from leaching, and increased mineral uptake by plant roots [27, 29, 77]. Moreover, Kh (HA) was more effective in improving plant growth, and this is due to the function of potassium in controlling many enzymes in plants [78, 79], and humate K (Kh or HA) also plays a great role as a biostimulant [80]. Several mechanisms function for plants under abiotic stresses to mitigate these stresses. One such mechanism is to enhance the activity of antioxidant enzymes such as catalase (CAT), peroxidase (POX), superoxide dismutase (SOD), and proline which play a very important role in scavenging reactive oxygen species (ROS) [47, 77, 80, 81].

Several previous studies have confirmed that HA (Kh) treatment increases the activity of antioxidant enzymes (CAT, SOD, and POX) under stress conditions and under salt stress conditions [47, 80, 82–85].

Salinity of irrigation water or the salinity of the soil in which the barley plant grows leads to a decrease in plant height, the number of spikes, the weight of 1000 grains, and the grain yield. But at the same time, total chlorophyll, relative water content, leaf osmotic potential, proline and potassium contents are considered as biochemical parameters in salt tolerance, [19, 20, 86, 87]. Many early studies confirmed that proline content and accumulation is related to osmotic tolerance and salt stress, in addition to being one of the most important physiological indicators of salt tolerance in barley, [88–95], as the concentration of proline increased in the tolerant genotypes studied relative to increasing salt stress and the increase was gradual with increasing stress [91, 93, 96].

Misra and Gupta [19] also found a positive correlation between the amount of free proline accumulation and salt tolerance as an indicator for determining salt tolerance potential among cultivars [97], whereas, the magnitude of the increase in free proline accumulation was higher in tolerant cultivars than in sensitive cultivars [19, 98].

Proline plays an important role in reducing the damaging effects of salt and in accelerating the repair processes following stress [99]. This is because proline is the only molecule among many compatible solutes that can act as a free radical scavenger and have antioxidant activity [100]. Then, proline is able to stabilize proteins and DNA as well as membranes [101]. It is suggested that the high proline concentration in salt-tolerant plants under salt stress may help maintain the structure and function of cellular macromolecules [97]. Many reports have also shown that proline acts as an osmo-protectant and is related to the tolerance mechanism under salt stress [94, 95, 102]. Besides being an osmolyte, proline confers enzymatic protection and increases membrane stability [103].

A number of studies have also found that proline accumulation related to the degree of salt tolerance and/or osmotic tolerance under treatment with humic acid and other treatments that increase plant tolerance to salinity [90–93, 104].

Our results obtained that, proline concentrations increased in plants treated with humic acid, as well as plants treated with fulvic acid, compared to the control treatment and also compared to those plants that were treated with the same fertilizer rate without the addition of humic acid or humic fulvic acid under salinity. This indicates that these treatments led to an increase in the tolerance of the barley plant to salinity and increased adaptation in a way better with salt stress and proline is a metabolite that enhances salt tolerance through osmotic adjustment [105, 106].

### Nutrient uptake and availability by barley plants

Under salinity conditions, important nutrients play an vital function in salinity stress tolerance in higher plants [107]. Nutrient availability may be affected by salinity stress and some nutrients may be insufficient or unavailable [108]. In the present investigation, N, P and K uptake were significantly increased by HA or FA treatment (Fig. 4 and Table 6). Treatment with organic amendments (HA or FA) significantly increased available nitrogen compared to control treatment, where treatment with HA and FA increased N availability, above the control treatment level, respectively. Thus, the treatment with organic amendments resulted in an increase in nitrogen uptake. From the results of this study, nitrogen uptake in grain and straw can be arranged as affected by the treatments in descending order T1 (HA + 100% RDP) > T2 (HA + 75% RDP) > T5 (FA + 100% RDP) > T3 (HA + 50% RDP) > and T6 (FA + 75% RDP). The treatment with organic amendments resulted in a significantly increased available potassium compared with the control treatment, which led to an increase in its uptake, whereas HA and FA increased the available K, respectively, over the control T12 (0% RDP). Therefore, K available as affected by the treatments can be arranged descending order T1 (HA + 100% RDP) > T2 (HA + 75% RDP > T5 (FA + 100% RDP) > T3 (HA + 50% RDP) > and T6 (FA + 75% RDP). The results included in (Fig. 4 and Table 6) showed that the uptake of N, P and K was significantly affected by the organic amendments (HA or FA). The treatment with the organic amendments under salinity conditions also led to a significantly increased available phosphorus compared with the control treatment, and thus increased its uptake by plants, whereas the application of HA and FA increased P uptake, respectively, above the control. Consequently, treatments can be arranged according to their effect on the amount of P uptake in grain and straw in downhill order as follows: T1 (HA + 100% RDP) > T2 (HA + 75% RDP) > T3 (HA + 50% RDP) > T5 (FA + 100% RDP) > T6 (FA + 75% RDP). Some studies showed that treatment with humic acid (Kh) mitigate the harmful effects of salinity by increase the absorption of elements, promoting plant growth [29], and stimulating the plant defense system against stress [47, 77].

The reason is to increase the absorption of elements by adding humic acid or adding fulvic acid, these organic matter play an important function in the soil ecosystem since they provide substrates for microbial decomposition, improve soil properties and increase its water-holding capacity, [38]. In the current study, the type of organic amendments tested, which include humic acid or fulvic acid, affected the availability and uptake of N, P and K, where humic acid was more effective than fulvic in the amount of elements uptake [22, 109, 110].

Previous studies have shown the importance of soil organic matter, as it represents the main original source of available nitrogen in the soil (N), in addition, it contains approximately 65% of the total soil phosphorous. It also provides significant amounts of sulfur (S) and other nutrients required for plant growth [111]. It has become established and well known the negative effect of salinity on plant growth and on the absorption and accumulation of elements in plant tissues [112]. It is well known that salinity leads to a deficiency in the uptakes of nutrients (N, P, K^+^, Ca, Mg and microelements), and high levels from Na and Cl to a lower of availability of microelements and their decrease in the rhizosphere [61]. In the present study, salinity decreased the K^+^ content in barley but the application of treatments increased content in grain and straw of barley (Fig. 4c and Table 6). A similar pattern of results was obtained, by Saidmuradi et al. [113] where they found that salinity stress reduced K^+^ uptake by plants, but maintaining adequate K^+^ levels in plants mitigated adverse effects of salinity [114, 115]. Under salinity stress condition, the content of Na^+^ in leaves decreased by application of Kh (HA) treatments and their combination which also led to increased K^+^ content in leaves, [3], (Fig. 4c and Table 6). Moreover, soil Kh (HA) application improved K uptake and reduced Na uptake in plant buds and shoots, [113]. Kh contains K^+^, which is known to be responsible for salinity tolerance, due to its competition with sodium in terms of binding and maintaining plant water status [116]. Na^+^ adsorbed by humic compounds as a result of Kh application also helps reduce Na content in shoots and allows more K^+^ uptake by roots [47, 117].

Finally, humic acid (HA), which is characterized as an essential component of soil, maintaining its health and maintaining its productive ability, retains water, organic dissolved atoms, binds mineral ions and sensitizes various soil reactions, stimulates plant growth, and bio-transforms pollutants [46, 118], as well as it raises the water-holding capacity of soil, which causes an increase in soil fertility [119, 120].

## Conclusions

From the results of this study, it can be concluded that, applying humic acid or fulvic acid and phosphorus fertilizer indicated that the effect of humic acid with is a good tool for increased nutrient availability, uptake and enhanced plant growth, and this may be the reason for increased salinity tolerance in barley to promotion barley growth and yield, particularly in saline soils. The findings illustrated that the combinations T1 (HA + 100% RDP), T2 (HA + 75% RDP), T5 (FA + 100% RDP), T3 (HA + 50% RDP), and T6 (FA + 75% RDP), resulted to improve most studied traits, and increased grain yield (Ton/ha), in the barley with the increase percentage 22.30, 16.42, 11.27, 10.78, and 7.11% in the first season, and 22.17, 16.63, 11.08, 10.84, and 6.99% in the second season, for grain yield compared with the recommended dose. Thus, it is recommended to treatment soil with humic acid with percentage of the RDP fertilizer at under salinity conditions, i.e., T1 (HA + 100% RDP), T2 (HA + 75% RDP), T3 (HA + 50% RDP) to increase growth and yield of the barley under saline conditions with saving in the phosphate fertilizer by percentage 25-50%.

### Supplementary Information

**Supplementary Material 1.** 

## Data Availability

The dataset supporting the conclusions of this article is included within the article.
