# Supplementary material for: Applications of humic and fulvic acid under saline soil conditions to improve growth and yield in barley
Source: BMC Plant Biol. 2024 Mar 15;24:191. doi: 10.1186/s12870-024-04863-6 (PMC10941484; doi:10.1186/s12870-024-04863-6)
Supplement: Supplementary file 1 — Supplementary Material 1. [file 12870_2024_4863_MOESM1_ESM.docx]

**Table S1.** Means monthly of climatic parameters during barely growth and development at the study site (2018/2019 and 2019/2020 seasons).

| Month | Air Temperature (oc) | | | | Relative Humidity (%) | | Precipitation (mm) | |
| --- | --- | --- | --- | --- | --- | --- | --- | --- |
|  | **Minimum** | | **Maximum** | |  |  |  |  |
|  | **2018/2019** | **2019/2020** | **2018/2019** | **2019/2020** | **2018/2019** | **2019/2020** | **2018/2019** | **2019/2020** |
| December | 12.7 | 11.2 | 25.9 | 22.7 | 33.3 | 30.1 | 1.08 | 0.62 |
| January | 11.4 | 10.0 | 24.5 | 19.8 | 45.4 | 41.7 | 2.07 | 2.24 |
| February | 10.1 | 9.3 | 22.7 | 21.2 | 43.5 | 40.5 | 5.35 | 5.78 |
| March | 12.9 | 11.2 | 24.3 | 23.2 | 42.9 | 43.7 | 0.65 | 0.51 |
| April | 13.7 | 15.5 | 25.2 | 26.1 | 50.8 | 50.6 | 0.00 | 0.00 |
